# Underestimated Prediabetic Biomarkers: Are We Blind to Their Strategy?

**DOI:** 10.3389/fendo.2022.805837

**Published:** 2022-03-07

**Authors:** Carla Luís, Raquel Soares, Pilar Baylina, Rúben Fernandes

**Affiliations:** ^1^ Laboratory of Medical & Industrial Biotechnology (LABMI)-Porto Research, Technology and Innovation Center (PORTIC), Porto, Portugal; ^2^ Departamento de Biomedicina, Faculdade de Medicina da Universidade do Porto, Porto, Portugal; ^3^ Instituto de Investigação e Inovação em Saúde (i3S), Universidade do Porto, Porto, Portugal; ^4^ ESS-IPP – Escola Superior de Saúde, Instituto Politécnico do Porto, Porto, Portugal

**Keywords:** diabetes, biomarkers, early diagnosis, galectin-3, ophthalmate, fetuin-A, prediabetes

## Abstract

Type 2 Diabetes (T2D) is currently one of the fastest growing health challenging, a non-communicable disease result of the XXI century lifestyle. Given its growing incidence and prevalence, it became increasingly imperative to develop new technologies and implement new biomarkers for early diagnosis in order to promote lifestyle changes and thus cause a setback of the disease. Promising biomarkers have been identified as predictive of T2D development; however, none of them have yet been implemented in clinical practice routine. Moreover, many prediabetic biomarkers can also represent potential therapeutical targets in disease management. Previous studies have identified the most popular biomarkers, which are being thoroughly investigated. However, there are some biomarkers with promising preliminary results with limited associated studies; hence there is still much to be understood about its mechanisms and associations in T2D pathophysiology. This work identifies and discusses the promising results of Galectin-3, Ophthalmate and Fetuin-A.

## Introduction

Type 2 Diabetes (T2D) is already considered a worldwide pandemic, no longer restricted to developed countries (1), responsible for more than 4.2 million deaths in 2019 (2) and a trigger for other non‐communicable diseases like cancer and cardiovascular diseases. It is a complex disease with different pathophysiology profiles concomitant with inflammation, adiposity/obesity, lipid oxidation, hyperglycemia, glycation/glycosylation, and oxidative stress, further discussed. Such mechanisms can lead to insulin secretion deficiency from pancreatic beta cells, tissue insulin resistance, and impaired compensatory response to insulin (3).

Pro-inflammatory signals lead to immune cell activation and recruitment, an immune response that can result in insulin resistance. Previous studies have detected that this likely happens due to phosphorylation in the insulin receptor substrate (4). A low state of chronic inflammation is found upon adipose tissue accumulation, particularly in the abdominal depot (5). Already well-established, the adipose tissue is an endocrine organ, metabolically active, responsible for numerous functions, hence releasing several factors (6). Such factors are dynamic intervenients in processes like food uptake (asprosin, leptin), inflammation (TNF-α, IL-1β, IL-6, resistin, among others), energy expenditure (visfatin), immunity (MCP-1, MIP-1α, among others), and metabolism homeostasis (UCP-1) (5, 6).

Common knowledge states that obesity is the imbalance between energy consumption and energy expenditure. When the expenditure rate is lower than the consumption rate, the accumulation in the fat deposits will increase, leading to obesity and consequently deregulated energy metabolism. A shift towards lipid oxidation to the detriment of glucose oxidation occurs (7) since fatty acids and glucose are the main substrates for oxidation. The Glucose/Fatty Acid Cycle (8) expresses the metabolic relationship between the two substrates. An increase in fatty acid levels leads to the inhibition of glucose oxidation and cell uptake, which can be stored as glycogen, resulting in hyperglycemia and, ultimately, in insulin resistance (9, 10).

Prolonged exposure to a hyperglycemic state also causes tissue damage by promoting glycation, where sugars are covalently bound to proteins without enzymatic intervention. These Amadori products produce complex irreversible glycation adducts, the so-called Advanced Glycation End products (AGEs), which are capable of damaging cells (like pancreatic beta cells), tissues, and processes (11). AGEs are also responsible for causing oxidative stress.

Oxidative stress state is a condition where the free radical production is higher than the antioxidant defense activity. The human defense to free radicals consists of two main mechanisms: the enzymatic domain, which includes catalase, glutathione peroxidase and reductase, and superoxide dismutase; and the non-enzymatic domain that includes antioxidants, such as vitamins – A, C and E, glutathione and uric acid (12).

Recently, metabolomic studies have been used to identify, quantify, and characterize the metabolites’ physicochemical properties of such pathways cascades, allowing a better comprehension of the overall metabolism (13). Identification of early alterations in the pathophysiological profiles of the prediabetic individual can be critical in order to break the diabetic cascade. Our aim is to identify promising biomarkers able to represent the metabolic alterations that occur during the pathophysiological transformation of prediabetes to the clinical stage of T2D.

A recent study performed a survey regarding review articles of the last decade identifying biomarkers (metabolites and microRNA) associated with prediabetes, impaired fasting glucose and impaired glucose tolerance (14). Interestingly enough, the most popular and characterized biomarkers aren’t yet implemented in the clinical practice. We believe that the present work could present and expose the underestimated prediabetic biomarkers to be academically embraced to a more extended comprehension. We identify Galectin-3, Ophthalmate and Fetuin-a, as encouraging metabolites to address, which have been neglected in the last 10 years.

## Galectin-3

**Figure 1:**
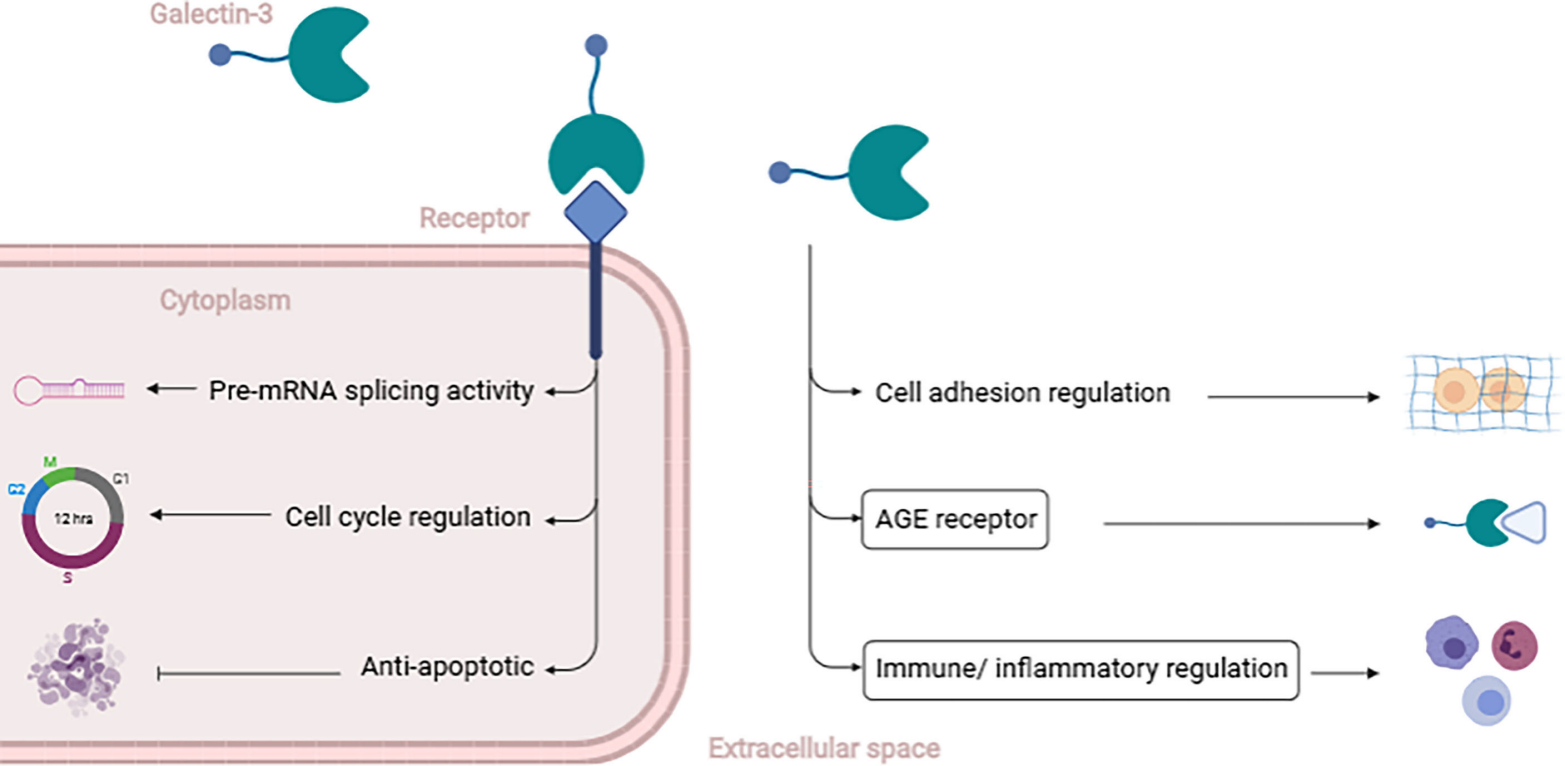
Brief description of Gal3**’**s functions. Gal3 exerts different functions depending on its location. Intracellularly, Gal3 can act as a Pre-mRNA splicing factor, regulating the cell cycle, promoting proliferation, and protecting from apoptosis. Extracellularly, Gal3 is able to regulate cell adhesion (either cell-to-cell or cell-to-matrix) and mediate features of T2D pathophysiology like binding to AGEs, regulating inflammatory and immune pathways (emphasized by the boxes) [20]. (Created with BioRender.com).

Galectin 3 (Gal3) is a member of an evolutionarily conserved family of soluble β-galactosidases binding lectins. It is known to be a relevant modulator of many biological functions (**Figure 1**) and an emerging player in the pathogenesis of common disease conditions, including cancer (15–17), immune/inflammatory diseases (18–20), and metabolic disorders like T2D (21–24).

In animal models, Gal3 is associated with increased oxidative status, a common feature in T2D. Our group has observed increased levels of Gal3 in diabetic mice (22), concomitant with higher concentrations of AGEs and 3-nitrotyrosine in the liver and kidney. Pejnovic et al. also described that Gal3 knockout mice revealed a higher Body Mass Index (BMI) and increased levels of inflammation on visceral adipose tissue. Therefore, aggravated inflammatory status on pancreatic islets associated with AGE accumulation which resulted in impaired glucose homeostasis (25).

In human studies, circulating Gal3 was elevated in obese individuals and negatively correlated with HbA1c in T2D patients. The connection between Gal3 and HbA1c is still not fully understood, although it is postulated that Gal3 may reduce oxidative stress by scavenging AGEs, which leads to lower levels of HbA1c (26). This association between Gal3 and the incidence of T2D was suggested in the Dallas Heart Study with logistic regression models. Results demonstrated that Gal3 levels were correlated with inflammation, subcutaneous adiposity, and insulin secretion, concordant with the previous results obtained in animal models (27). Moreover, as a prediabetic biomarker, a study performed in First Degree Relatives of subjects with T2D observed that Gal3 is correlated with increased waist circumference, HbA1c, glucose, and high-sensitivity C-reactive protein levels and can also be determinant for the prediction of diabetes (28).

## Ophthalmate

Ophthalmate (OPH) or ophthalmic acid belongs to the class of organic compounds known as oligopeptides isolated from the crystalline lens. It is a tripeptide analog of glutathione (GSH), composed of glutamate and glycine residues as GSH, but where GSH cysteine residue is replaced by α-aminobutyrate (α-AB) (29). GSH is synthesized in two steps, first glutamate and cysteine produce γ-glutamylcysteine catalyzed by γ-glutamylcysteine synthetase. Then, glycine by glutathione synthetase is added, and GSH is produced (30), having a crucial role in the antioxidant metabolism. The limiting step of GSH synthesis is the low cysteine levels produced exclusively by the transsulfuration pathway in conjugation with the methionine metabolism (31).

**Figure 2:**
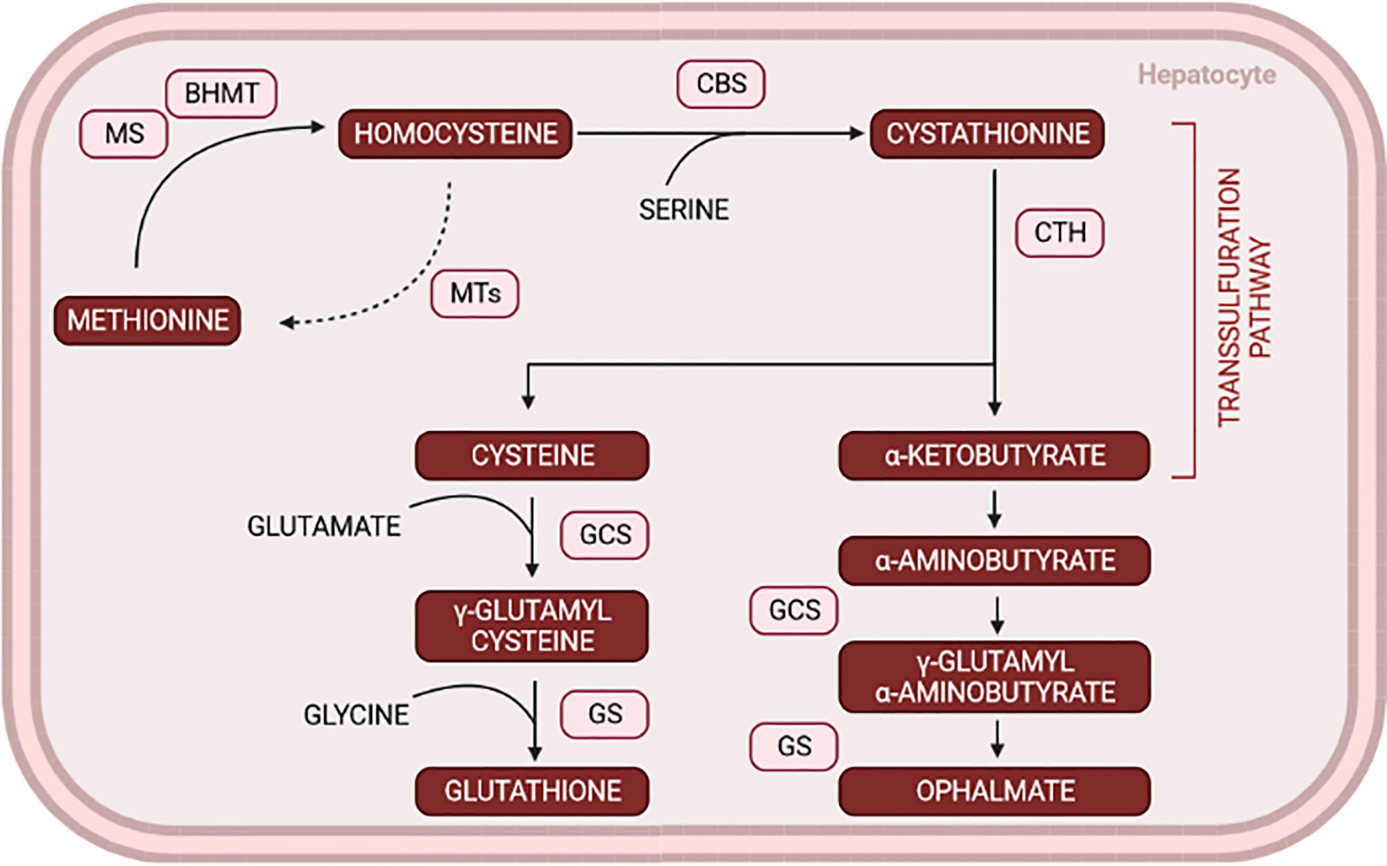
Glutathione and ophthalmate shared pathways *via* transsulfuration in conjugation with methionine metabolism. The increase in OPH levels works as a rather compensatory mechanism when glutathione depletion occurs. MS, methionine synthase; BHMT, Betaine homocysteine methyltransferase; MTs, methyltransferases; CBS, cystathionine β-synthase; CTH, cystathionase; GCS, glutamyl-cysteine synthase; GS, Glutathione synthetase. (Created with BioRender.com).

Studies performed in mice showed that overall oxidative stress related to T2D might lead to the depletion of glutathione, which motivates the depletion of cysteine and, consequently, activates the ophthalmate synthesis (32). In OPH, cysteine is replaced by α-AB, a contributor metabolite of several pathways, including amino acid catabolism (30) (**Figure 2**). Therefore, OPH levels are inversely correlated with GSH concentration. Given that GSH quantification is unstable, therefore unreliable due to auto-oxidation, by lacking the reducing cysteine moiety, OPH could represent a more accurate biomarker due to its stability.

Soga and colleagues established a method to determine OPH levels in response to oxidative stress in animal models. They used different compounds to deplete GSH levels and observed that most of the metabolites detected in the liver were also detected in the serum and that ophthalmate increased 5-fold 1h after treatment concomitantly with a GSH decrease. These results, among others (30, 33), can help validate OPH as a biomarker and evaluate therapeutical assessments, potentially helping in the early detection of several diseases associated with oxidative stress like T2D (32).

Metabolomic studies including OPH are being performed in humans with positive insights, but not in prediabetes (34). Hence, much more data is needed, like cutoffs, quantification methods, and full comprehension of the limitations and benefits to implement OPH as a prediabetic biomarker.

## Fetuin-a

**Figure 3:**
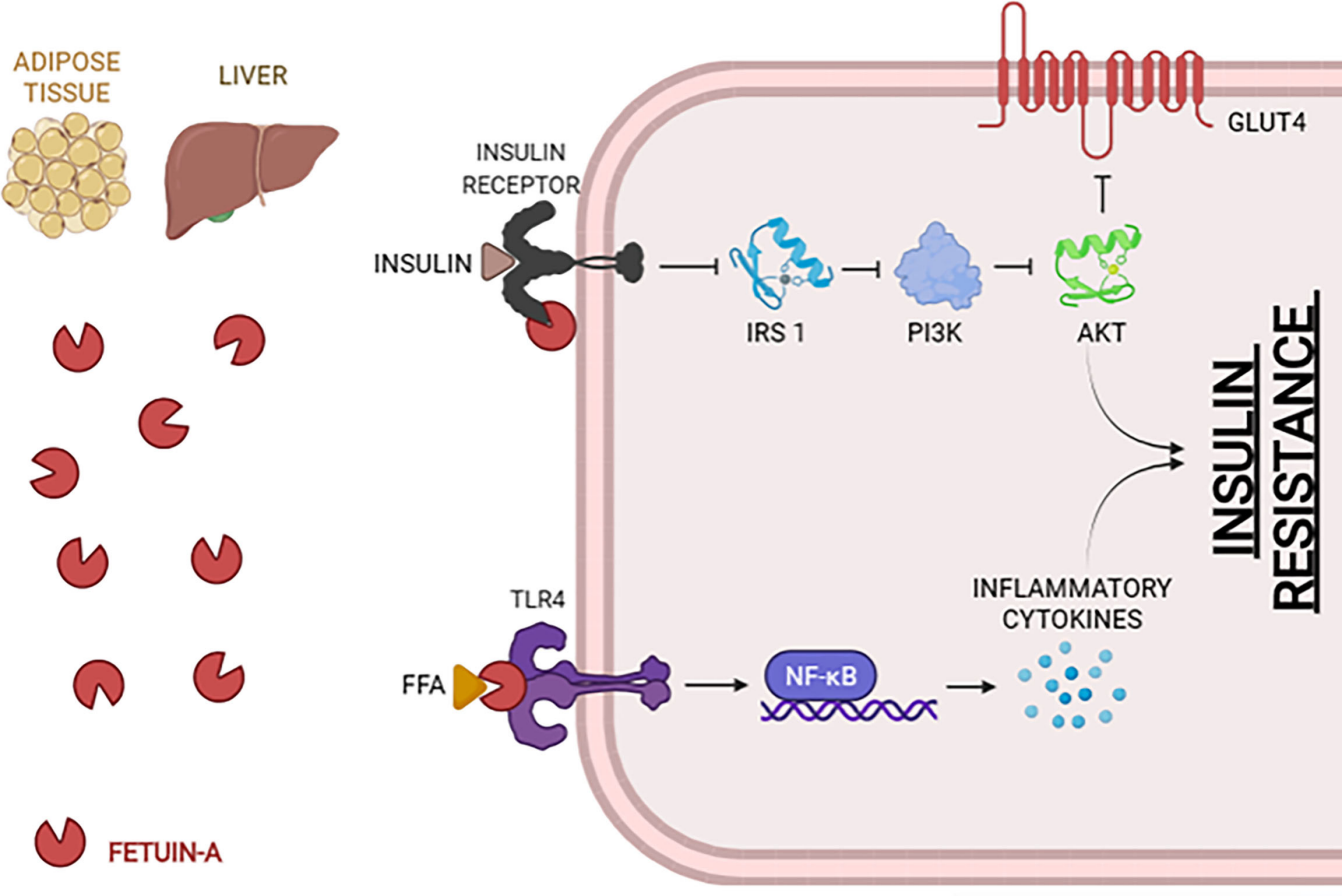
Mechanisms of Fetuin-A in order to promote insulin resistance. The association of Fetuin-A with the insulin receptor inhibits IRS1 and, therefore, the cascade that will block GLUT4. *Via* TLR4, Fetuin-A will activate the NF-kB genes, which will promote the secretion of inflammatory cytokines and lead to insulin resistance. FFA, Free Fatty Acids; NF-kB, Nuclear Factor Kappa-light-chain-enhancer of activated B cells; IRS1, Insulin Receptor Substrate 1; PI3K, Phosphoinositide 3-Kinases; AKT, Protein kinase B; GLUT4, Glucose transporter type 4. (Created with BioRender.com).

Fetuin-A is a 64-kDa glycoprotein secreted by the adipose tissue and the liver, associated with several metabolic functions and pathological progressions in insulin resistance, vascular calcification, cell dysfunction, among others (35, 36). Fetuin-A is increased in pathologies like T2D, metabolic syndrome, obesity, and obesity-related complications such as non-alcoholic fatty liver disease and chronic kidney disease. Fetuin-A inhibits the insulin receptor through tyrosine phosphorylation, the capture of macrophages, and TLR4 activation by acting as an endogenous ligand and activating NF-kB and triacylglycerol accumulation (**Figure 3**) (35, 37, 38).

In the case-cohort study performed by Stefan et al. in 2008 (39), a considerable number of individuals (n= 2500) were randomly selected within the European Prospective Investigation into Cancer and Nutrition (EPIC)-Potsdam study. Of the 2500 individuals, 2,164 were nondiabetic at baseline. After 7 years follow-up, a total of 703 were subject to analysis. The results indicated that Fetuin-A was an independent risk factor for Type 2 Diabetes. Circulating Fetuin-A levels remained positively correlated to diabetic risk even after adjustment for sex, BMI, waist circumference, and lifestyle risk factors (39).

A study performed in eastern India by Dutta and colleagues included the quantification of Fetuin-A in 2119 individuals. The main observation was that progressors had higher levels of Fetuin‐A (40). The same observation was described in another case-cohort, the Nurses’ Health Study (NHS; 470 cases), where individuals with higher levels of Fetuin-A had the highest risk of developing diabetes (41).

Though in a more advanced investigation stage than the other biomarkers, Fetuin-A has revealed some controversial results regarding the association with diabetic complications like cardiac pathologies (42, 43).

## Discussion and Conclusion

Type 2 Diabetes is a complex pathology with high prevalence and morbidity rates. We believe that the identification of new metabolites able to translate diabetic progression will significantly decrease the prevalence of T2D. Our present work focused on potential metabolites with limited associated studies but with promising results.

The background knowledge was a previous work which identified the most popular biomarkers, the most likely to endorse the diagnostic clinical routine in T2D prevention (14). We recognized that such could be, by itself, a limitation to our study. Moreover, due to the nature of the publication, we did not assess publication bias or strength of evidence of the included articles. To present a more embracing review, we summarize our study in **Table 1**.

**Table 1:** Description of the main results of original articles of Galectin-3; Ophthalmate; Fetuin-A in animal and clinical studies.

GALECTIN-3	
Animal Studies	Clinical Studies
A prediabetic mouse model was used to evaluate the effect of piperine. It was observed a reduction in Gal3 and an improvement of beta-cell dysfunction (22). Study of renal function and prediabetic mouse model observed higher levels of Gal3 in the proximal tubules and glomeruli of HFD mice and attenuated by Adam17 depletion (45). Diabetic mouse model presented higher levels of Gal3 in liver and kidney and Gal3 was correlated with oxidative stress, specifically with AGE products and 3NT (22). Male Gal-3–deficient mice demonstrated that animals fed with HFD revealed an increased in BMI, adipose tissue, FBG, insulin resistance and inflammation levels when compared to a ND (25).	Gutenberg Health Study cohort evaluated Gal3 levels in individuals with normoglycemia, prediabetes and T2DM with 5 years follow-up. Gal3 was associated with cardiovascular function but not with cardiovascular or all-cause deaths in prediabetic subjects (46). Individuals subjected to a carrageenan-free diet presented decreased levels of HbA1c, HOMA-IR, IL-8 and Gal3 (47). Gal3 levels were higher in the diabetic group. Also, Gal3 was positively correlated with hs-CRP, L-NMMA, HbA1c, neopterin and FPG (48). Levels of Gal3 were evaluated in the Dallas Heart Study. It was observed a correlation between Gal3 and hs-CRP, IL-18, MCP-1, TNF-R1A, myeloperoxidase, C-peptide and subcutaneous adiposity (27). Not only is Gal3 associated with subjects with T2D but also with their first-degree relatives. Correlation between Gal3 and waist circumference, HbA1c, glucose and hs-CRP was found (28) Exercise training did not reduce concentration of Gal3 in prediabetic individuals although cardiorespiratory fitness was observed to be negatively correlated with Gal3 Levels (49). A study with 3 groups (Control, Prediabetes and diabetes) measure Gal3 and found a correlation with FPG, 2hPG, CRP and HOMA-IR. Diabetic group presented the higher levels of Gal3 (50). Gal3 levels in patients with insulin resistance were correlated with GDR, fasting insulin, adiponectin, hs-CRP, IL−10 and IL-6 (23) .
**OPHTHALMATE**	
**Animal Studies**	**Clinical Studies**
Ophthalmate is considered an oxidative stress biomarker indicating Glutathione depletion in hepatic and serum levels (32). C57BL/6N mice had increased levels of OPH and decreased Glutathione concentration after fasting (30).	Not available
**FETUIN-A**	
**Animal Studies**	**Clinical Studies**
Wistar rats were used as controls and HHT rats were used as obese prediabetic model and subject to empagliflozin. With treatment, Fetuin-A decrease alongside with improved insulin sensitivity and decrease inflammation (51). Non-obese TLR knockout mouse is a prediabetic model by TLR-4 deficiency diabetes-promoted. Fetuin-A is independent of TLR-4 expression but can act as an adapter in the FFA-TLR-4 interaction (52). In a type 1 non-obese diabetic mouse, a prediabetic state was already found in the liver. The transition to a diabetic state was accompanied by increased levels of Fetuin-A (53).	T2D subjects had elevated Fetuin-A when compared to normoglycemia subjects. Fetuin-A was also associated with diabetic risk factors. Baseline levels of Fetuin-A independently contributed to the progression of prediabetes to T2D (54). Fetuin-A is capable of crossing the blood-brain barrier and is associated with depression regardless of insulin sensitivity status (55). Circulating levels of Fetuin-A alongside with Oxytocin were found to be decreased in subjects with MetS and with MetS plus prediabetes when compare to control (56). A study in pregnant women with previous gestational dysglicemia with a 3-year follow up. Results observed that circulating Fetuin-A record the state of beta cell function and insulin sensitivity (57). A study in prediabetic individuals evaluated Neck Circumference and Neck-Height Ratio as biomarkers in NAFLD. Results showed that high levels of Fetuin-A is associated with highest NHtR, higher glycemic levels, insulin resistance and dyslipidemia. Also, Fetuin-A can be a predictor of liver stiffness in prediabetes (58). Fetuin-A (and Adiponectin) were independent predictors of diabetic progression in the following cohorts: European Prospective Investigation into Cancer and Nutrition – Potsdam study and the Nurses' Health Study (41) Evaluation of diabetic progression in subjects with prediabetes observed that diabetic progressors revealed higher levels of Fetuin-A (40). Circulating Fetuin-A was evaluated in prediabetic individuals with or without NAFLD. Levels of Fetuin-A were increased in the NAFLD group (59).

2hPG - 2-hour Post Plasma Glucose; 3NT – 3 NitroTyrosine; AGE – Advanced Glycation Endproducts; BMI – Body Mass Index; FBG – Fasting Blood Glucose; FFA – Free Fatty Acids; FPG – Fasting Plasma Glucose; Gal3 – Galectin-3; GDR – Glucose Disposal Rate; HFD – High Fat Diet; hs-CRP – high-sensitivity C-reactive protein; L-NMMA - NG-monomethyl L-arginine; MetS – Metabolic Syndrome; NAFLD – Nonalcoholic Fatty Liver Disease; ND – Normal Diet; NHtR – Neck Height Ratio; TLR – Toll Like Receptor;

Survey performed in PubMed, Scopus and Google Scholar regarding each biomarker and their association with prediabetes/diabetes in the last ten years.

All displayed biomarkers have a correlation with one or more pathways diabetes-associated such as oxidative stress, insulin resistance, and impaired glucose metabolism. Galectin-3 is associated with pathologies connected to fibrosis, inflammation and oxidative stress. Ophthalmate associated with glutathione metabolism, and therefore oxidative stress and Fetuin-A is associated with calcification, inflammation, and insulin resistance. A broad analysis of the three biomarkers (**Table 1**) uncover that ophthalmate is still in an early research stage. However, Galectin-3 and Fetuin-A are already under investigation in human cohorts, evidencing the potential to become new biomarkers of prediabetes.

The main objective of our study was to assess the importance of underestimated biomarkers and expose the results obtained until now. The importance of implementing new screening methods in routine clinical practice will definitely improve prediabetes diagnosis. An early diagnosis will allow for well-timed lifestyle alterations, minimizing pharmacological drugs and alleviating national health systems. Moreover, tracking metabolome alterations will unravel new therapeutical targets. A controlled hyperglycemic status will help avoid other diabetes-associated comorbidities, like cardiovascular, neuropathy, nephropathy, retinopathy diseases, among others.

## Author Contributions

Conceptualization: RF and PB. Writing—original draft preparation: CL. Writing—review and editing: CL, PB, RS, and RF. Supervision: RF and RS. All authors contributed to the article and approved the submitted version.

## Funding

This work was supported by FCT – Fundação para a Ciência e Tecnologia (REF UID/BIM/04293/2013) and by the following scholarships: (Ref. SAICT2016/FEDER/BIO4DIA/BTI) and (SFRH/BD/146489/2019).

## Conflict of Interest

The authors declare that the research was conducted in the absence of any commercial or financial relationships that could be construed as a potential conflict of interest.

## Publisher’s Note

All claims expressed in this article are solely those of the authors and do not necessarily represent those of their affiliated organizations, or those of the publisher, the editors and the reviewers. Any product that may be evaluated in this article, or claim that may be made by its manufacturer, is not guaranteed or endorsed by the publisher.
